# Perfusion Index as a Predictor of Hypotension Following Spinal Anesthesia in Below-Umbilical Surgeries

**DOI:** 10.7759/cureus.116215

**Published:** 2026-09-14

**Authors:** Praneshwori Sinam, Priyanka Dev, Khumanthem M Devi, Laltanpuii Sailo, Rajani Thabah, Nari Lyngdoh, Neha Rawat

**Affiliations:** 1 Anesthesiology and Critical Care, North Eastern Indira Gandhi Regional Institute of Health and Medical Sciences, Shillong, IND; 2 Transfusion Medicine, North Eastern Indira Gandhi Regional Institute of Health and Medical Sciences, Shillong, IND

**Keywords:** diagnostic accuracy, haemodynamic monitoring, hypotension, perfusion index, pulse oximetry

## Abstract

Background

Hypotension following spinal anesthesia is common and may adversely affect end-organ perfusion. Perfusion index (PI), derived non-invasively from pulse oximetry, reflects peripheral vascular tone and may help identify patients at risk of post-spinal hypotension. This study aimed to determine the diagnostic threshold of baseline PI for predicting hypotension following spinal anesthesia in adults undergoing elective below-umbilical surgery and to assess its diagnostic performance.

Methods

This prospective observational diagnostic accuracy study included adult patients aged 18-65 years with the American Society of Anesthesiologists physical status I or II undergoing elective below-umbilical surgery under spinal anesthesia. Baseline PI, heart rate, and blood pressure were recorded before spinal anesthesia, followed by serial hemodynamic and PI measurements for 30 minutes. Hypotension was defined as a decrease in mean arterial pressure of >20% from baseline. Receiver operating characteristic analysis was performed to determine the optimal baseline PI cut-off using Youden's index, with an estimation of sensitivity, specificity, predictive values, and accuracy.

Results

A total of 92 patients were analyzed, of whom 21 (22.8%) developed post-spinal hypotension. Baseline PI was significantly higher among patients who developed hypotension than among those who did not (4.38±1.67 vs. 1.51±0.53, P<0.001). Baseline PI demonstrated excellent discriminatory ability, with an area under the receiver operating characteristic curve of 0.999 (95% confidence interval: 0.995-1.000, P<0.001). A baseline PI of ≥2.53 yielded 100% sensitivity, 98.6% specificity, 95.2% positive predictive value, 100% negative predictive value, and 98.9% overall accuracy.

Conclusion

Baseline PI demonstrated excellent diagnostic performance for predicting post-spinal hypotension in adults undergoing elective below-umbilical surgery. A baseline PI of ≥2.53 may help identify patients at increased risk and facilitate anticipatory hemodynamic management.

## Introduction

Spinal anesthesia is widely used for below‑umbilical surgeries as it avoids airway manipulation and allows early recovery, but hypotension from sympathetic blockade and reduced vascular resistance remains a common concern that can compromise organ perfusion and patient comfort [1]. Perfusion index (PI) is a simple, non-invasive value derived from the pulse oximeter that indirectly reflects peripheral vascular tone and microcirculatory perfusion. Higher baseline PI indicates relative vasodilation and lower vascular tone, which may predispose to a larger fall in blood pressure after spinal anesthesia [1]. Several studies in lower abdominal and cesarean surgeries have shown that baseline PI correlates with the incidence of hypotension following subarachnoid block and that receiver operating characteristic (ROC)‑derived cut‑off values can predict patients at risk of hypotension with good diagnostic performance [2-5]. However, most data are from specific groups, such as parturients, and evidence from a broader adult population undergoing below-umbilical surgeries is limited [6-7].

The primary objective of this study was to determine the threshold value of baseline PI for diagnosing hypotension following spinal anesthesia in adult patients undergoing below‑umbilical surgeries. The secondary objectives were to assess the sensitivity and specificity of PI in predicting hypotension and to study its association with the incidence of hypotension after spinal anesthesia in this population.

## Materials and methods

Study design and setting

This prospective observational diagnostic accuracy study was conducted in the Department of Anesthesiology at a tertiary care center in North-East India over a period of one year (from November 2024 to December 2025).

Study population

Patients aged 18-65 years of either sex, classified as American Society of Anesthesiologists (ASA) physical status I or II, and scheduled for elective below-umbilical surgery under spinal anesthesia were eligible for inclusion. Patients were excluded if they had significant systemic disease involving the cardiac, hepatic, or renal systems; septicemia; peripheral vascular disease; coagulation disorders or were receiving anticoagulant therapy; local infection at the proposed site of spinal anesthesia; spinal deformity; a history of allergy to local anesthetic agents; baseline PI <0.2 or >10; or refused to provide informed consent.

Sample size and sampling

All eligible participants constituted a single study cohort and were recruited using purposive sampling. The sample size was calculated based on a previously reported sensitivity of 94.21% for PI in predicting hypotension [2]. Assuming an absolute precision of 5% at a 95% confidence level and allowing for a 10% non-response or attrition rate, the required sample size was calculated to be 94 participants.

Index test and baseline assessment

The index test was baseline PI, measured before the administration of spinal anesthesia. After enrolment, all participants were preloaded with 500 mL of Ringer's lactate intravenously over 20 minutes. Standard monitoring, including electrocardiography, non-invasive blood pressure, and pulse oximetry, was instituted.

PI was measured with participants in the supine position using a pulse oximeter probe (Masimo Medical Technologies, India) attached to the right index finger to maintain uniformity of measurement. Baseline PI, heart rate (HR), systolic blood pressure, diastolic blood pressure, and mean arterial pressure (MAP) were recorded before spinal anesthesia.

Spinal anesthesia

A subarachnoid block was performed under strict aseptic precautions by an anesthesiologist who was blinded to the baseline PI measurement. A 25-gauge Quincke needle was inserted at the L3-L4 or L4-L5 intervertebral space. After confirming free flow of cerebrospinal fluid, 3 mL (15 mg) of 0.5% hyperbaric bupivacaine was administered intrathecally. The patient was positioned supine immediately following the injection. Patients who did not achieve T6 level sensory block were excluded from the study.

Reference outcome and hemodynamic monitoring

The target condition was the development of hypotension following spinal anesthesia, defined as a decrease in MAP of >20% from the individual baseline MAP. This criterion was used as the reference outcome against which the diagnostic performance of baseline PI was assessed. PI, HR, and blood pressure were recorded at two-minute intervals for the first 20 minutes following administration of spinal anesthesia and subsequently at five-minute intervals for the next 10 minutes. When hypotension occurred, intravenous mephentermine 6 mg was administered as a bolus and repeated as required according to the clinical response. Bradycardia, defined as HR <55 beats/minute, was treated with intravenous atropine 0.6 mg. Patients requiring conversion to general anesthesia were withdrawn from the study.

Data collection

Data were collected prospectively using a structured data collection format. Variables recorded included demographic characteristics, ASA physical status, baseline PI, serial PI measurements, HR, systolic blood pressure, diastolic blood pressure, MAP, and occurrence of hypotension following spinal anesthesia.

Statistical analysis

Data were entered and analyzed using IBM SPSS Statistics for Windows Version 29 (IBM Corp., Armonk, NY, USA). Continuous variables were expressed as mean ± standard deviation, while categorical variables were presented as frequencies and percentages. Appropriate statistical tests were used for comparison between participants who developed hypotension and those who did not, based on the type and distribution of the variables.

The diagnostic performance of baseline PI (index test) for predicting the occurrence of hypotension (target condition) following spinal anesthesia was assessed using ROC curve analysis. The area under the curve (AUC) with its 95% confidence interval was calculated to assess the discriminatory ability of baseline PI. The optimal baseline PI cut-off value was determined using Youden's index. At the identified cut-off, sensitivity, specificity, positive predictive value, and negative predictive value were calculated. A two-sided P-value of <0.05 was considered statistically significant.

Ethical considerations

The study was approved by the Institutional Ethics Committee of North Eastern Indira Gandhi Regional Institute of Health and Medical Sciences (Approval No. NEIGR/IEC/M2/F2/2024, dated 20 August 2024). Written informed consent was obtained from all participants before enrolment. Participation was voluntary, and participants were informed about the purpose and procedures of the study. Confidentiality and anonymity of participant information were maintained throughout the study.

## Results

A total of 92 patients undergoing elective below‑umbilical surgeries under spinal anesthesia were analyzed out of 94 assessed for eligibility (Figure 1).

**Figure 1:**
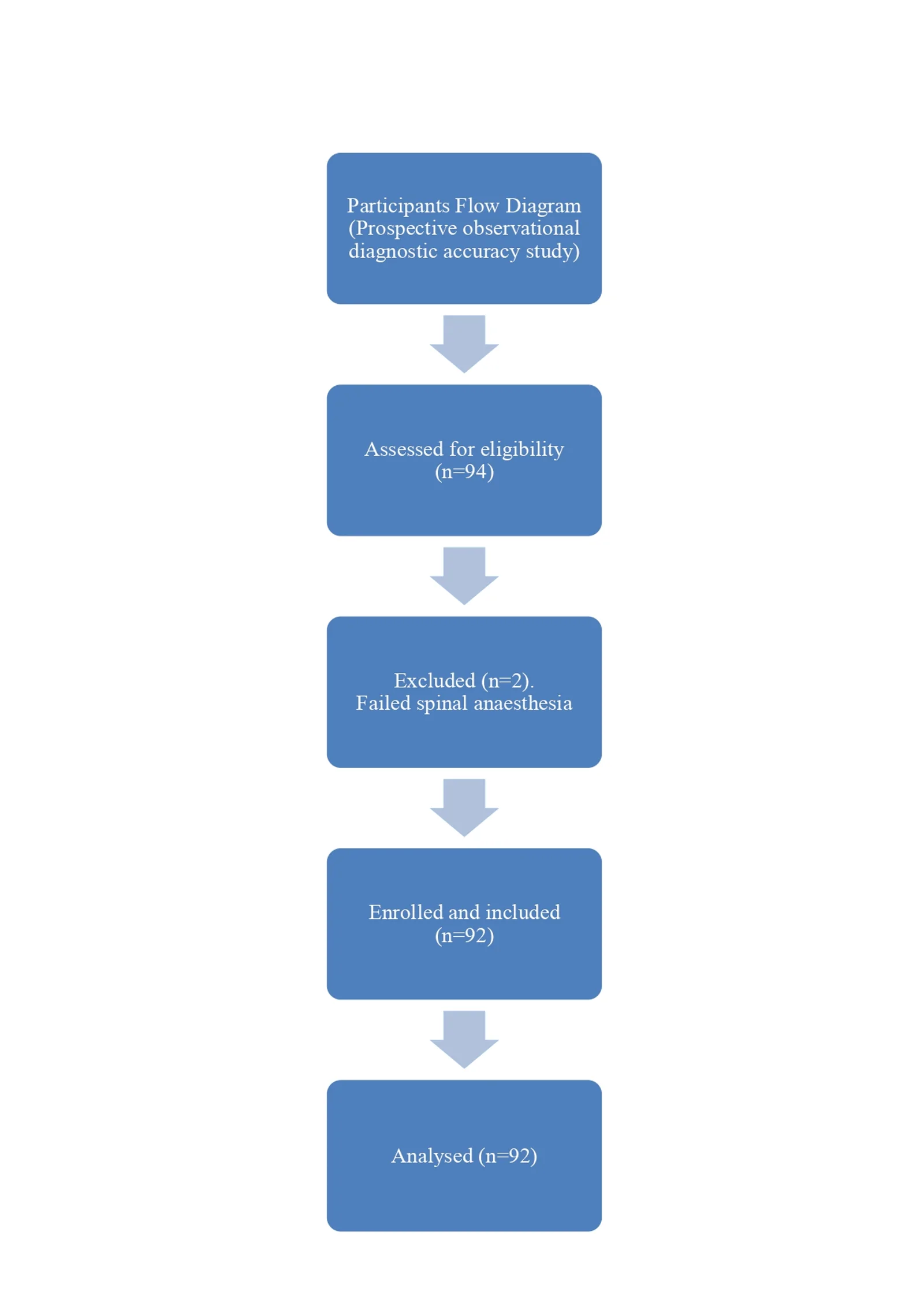
Flow diagram of the participants The flow diagram depicts participant selection and inclusion in the prospective observational diagnostic accuracy study.

The mean age was 38.61±12.84 years, 56.5% were male, and the mean BMI was 23.84±4.71 kg/m²; majority of the patients were from the general surgery department (61%), and most patients belonged to ASA physical status I (77.2%). Post‑spinal hypotension occurred in 21 (22.8%) patients, while 71 (77.2%) patients did not develop hypotension (Table 1).

**Table 1:** Baseline characteristics of the study participants (N=92). Data are presented as n (%) unless otherwise specified. *Continuous variables are expressed as mean ± SD. BMI, body mass index; ASA, American Society of Anesthesiologists physical status; SD, standard deviation

Baseline characteristics	n (%)
Age (years)*	38.61 ± 12.84
Gender	
Male	52 (56.5)
Female	40 (43.5)
BMI (kg/m^2^)*	23.84 ± 4.71
Types of the surgery	
General surgery	56(61)
Orthopedic surgery	24(26)
Gynecological surgery	12(13)
ASA	
Class I	71 (77.2)
Class II	21 (22.8)
Hypotension after spinal anesthesia	
Present	21 (22.8)
Absent	71 (77.2)

Baseline demographic and clinical characteristics were comparable between patients with and without post‑spinal hypotension. There were no statistically significant differences between the hypotension and no‑hypotension groups with respect to age, gender distribution, BMI, ASA class, baseline HR, or baseline MAP (all P>0.05). However, baseline PI was significantly higher in patients who developed hypotension compared with those who did not (4.38±1.67 vs 1.51± 0.53, P=0.000) (Table 2).

**Table 2:** Comparison of baseline characteristics between patients with and without post-spinal hypotension(N=92) Data are presented as n (%) unless otherwise specified. *Continuous variables are expressed as mean ± SD. Student's t-test was used for continuous variables and the chi-square (χ²) test for categorical variables. A P-value of <0.05 was considered statistically significant. BMI, body mass index; ASA, American Society of Anesthesiologists physical status; HR, heart rate; MAP, mean arterial pressure; PI, perfusion index

Characteristics	Hypotension, n (%)	No hypotension, n (%)	t/chi-square value	P-value
Age (years)*	39.85 ± 10.57	38.26 ± 13.45	t=0.490	0.628
Gender				
Male	12 (23.1)	40 (76.9)	χ²=0.126	0.571
Female	8 (20.0)	32 (80.0)		
BMI (kg/m^2^)*	24.66 ± 5.65	23.61 ± 4.44	t=0.880	0.380
ASA				
Class I	13 (18.3)	58 (81.7)	χ²=2.150	0.143
Class II	7 (33.3)	14 (66.7)		
Baseline HR*	82.75 ± 19.55	86.88 ± 15.53	t=0.990	0.324
Baseline MAP*	105.25 ± 9.66	102.06 ± 12.36	t=1.070	0.289
Baseline PI*	4.38 ± 1.67	1.51 ± 0.53	t=13.100	0.000

Correlation analysis between PI and hemodynamic variables at different time points showed a significant negative correlation between PI and MAP at several intervals after spinal anesthesia, particularly from 4 to 25 minutes, indicating that higher PI values were associated with lower MAP during this period (Figure 2).

**Figure 2:**
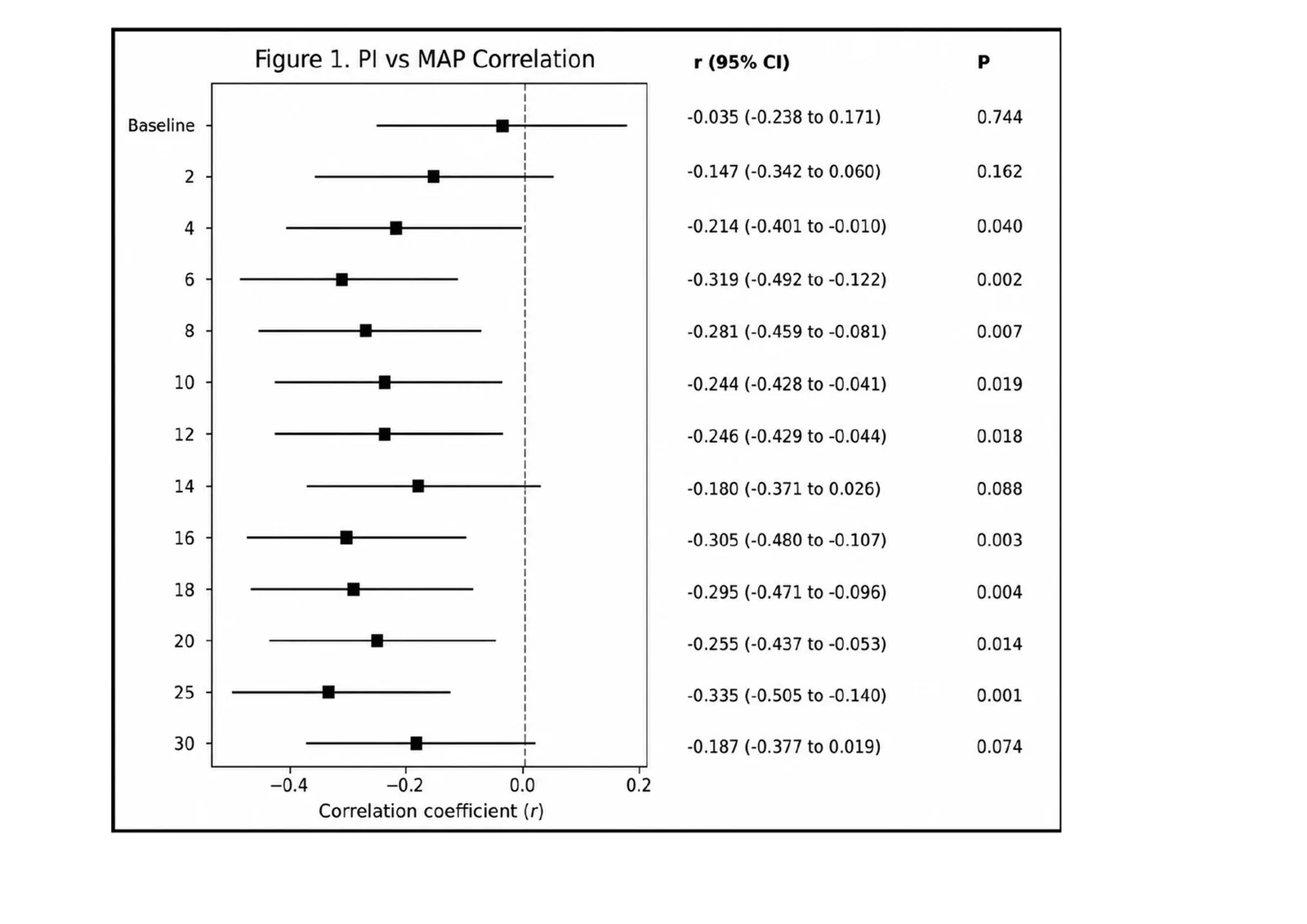
Correlation between PI and MAP following spinal anesthesia at different time points (N=92) The figure presents the correlation coefficient (r) with 95% CIs at baseline and at successive time points after spinal anesthesia. Negative correlation coefficients indicate an inverse relationship between PI and MAP. The dashed vertical line represents r= 0 (no correlation). P<0.05 was considered statistically significant. PI, perfusion index; MAP, mean arterial pressure; CI, confidence interval

The correlations between PI and HR at corresponding time points were weaker and did not reach statistical significance at most intervals (Figure 3).

**Figure 3:**
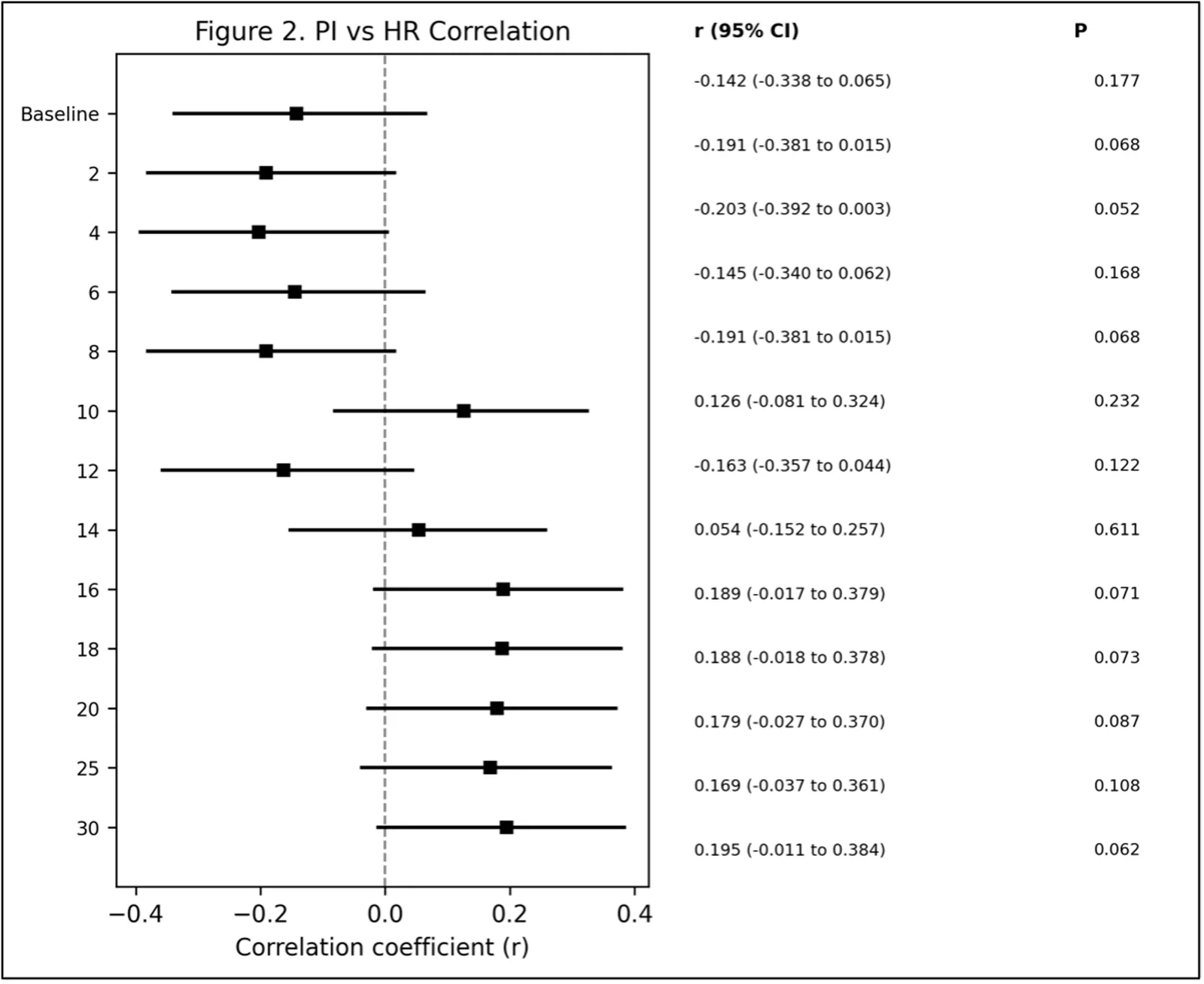
Correlation between PI and HR following spinal anesthesia at different time points (N=92) The figure presents the correlation coefficient (r) with 95% CIs between PI and HR at baseline and at successive time points after spinal anesthesia. Positive correlation coefficients indicate a direct relationship, whereas negative correlation coefficients indicate an inverse relationship between PI and HR. The dashed vertical line represents r=0 (no correlation). P<0.05 was considered statistically significant. PI, perfusion index; HR, heart rate; CI, confidence interval

ROC analysis for baseline PI in predicting post‑spinal hypotension demonstrated excellent diagnostic performance. The AUC was 0.999 (95% CI 0.995-1.000, P=0.000), indicating near‑perfect discrimination between patients with and without hypotension (Figure 4, Table 3).

**Figure 4:**
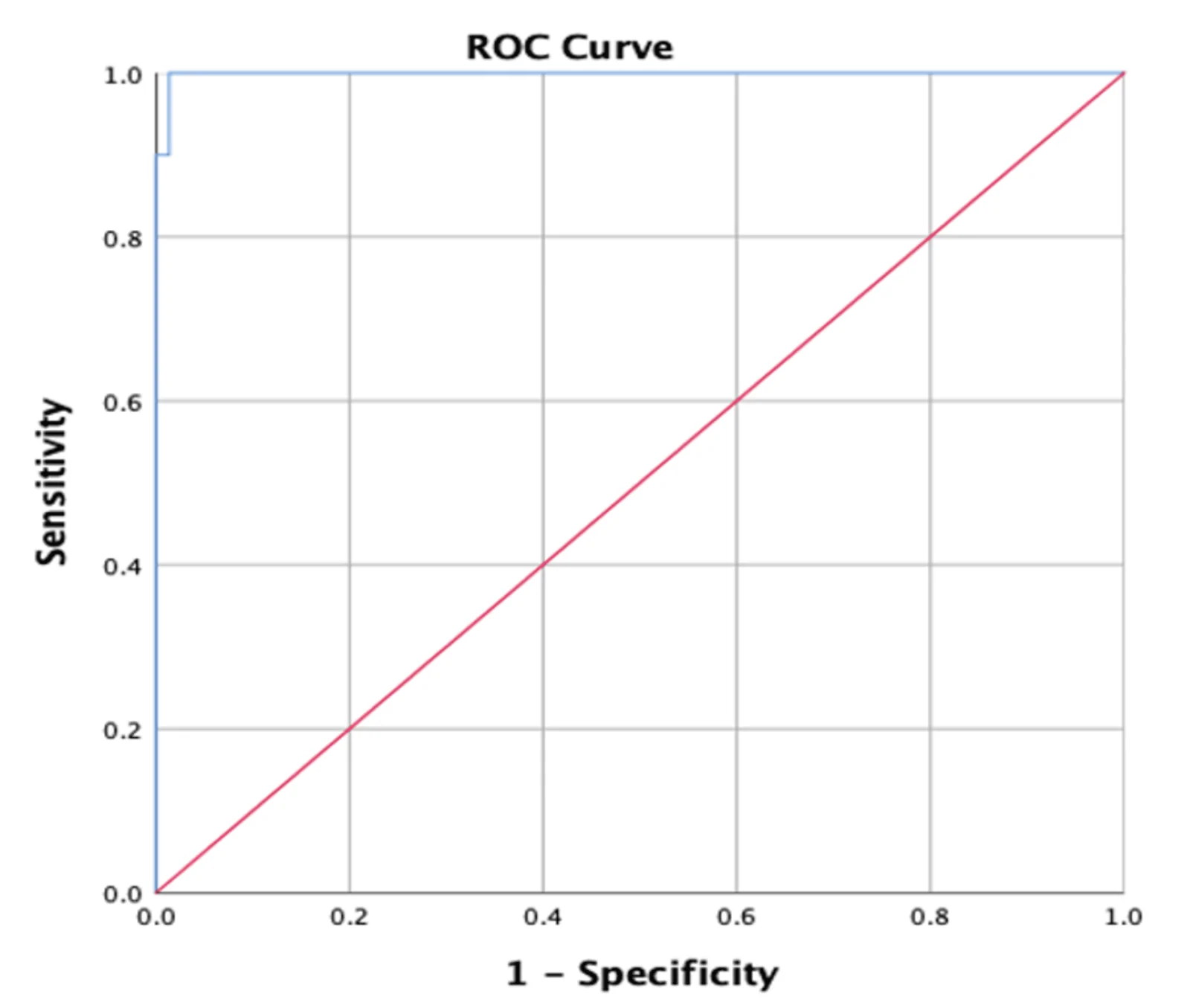
ROC curve of baseline PI for predicting post-spinal hypotension The ROC curve demonstrates the diagnostic performance of baseline PI in identifying patients who developed post-spinal hypotension. The y-axis represents sensitivity, and the x-axis represents 1-specificity. The diagonal reference line represents no discriminatory ability (AUC = 0.50). An AUC closer to 1 indicates better discriminatory ability. ROC, receiver operating characteristic; PI, perfusion index; AUC, area under the curve

**Table 3:** Diagnostic performance of baseline PI for predicting hypotension following spinal anesthesia. The AUC is presented with its Std. Error, P-value, and 95% CI. An AUC closer to 1.0 indicates better discriminatory ability. A P-value of <0.05 was considered statistically significant. Std. Error, standard error; PI, perfusion index; AUC, area under the curve; CI, confidence interval

Area	Std. Error	P-value	95% confidence interval	
			Lower bound	Upper bound
0.999	0.002	0.000	0.995	1.000

The optimal baseline PI cut‑off value identified by maximizing Youden’s index was ≥2.53, which yielded a sensitivity of 100.0%, specificity of 98.6%, positive predictive value of 95.2%, negative predictive value of 100.0%, and an overall accuracy of 98.9% for predicting post‑spinal hypotension (Table 4).

**Table 4:** Diagnostic performance of baseline PI at the optimal cut-off for predicting hypotension following spinal anesthesia The cut-off value was ≥2.530. Diagnostic performance is expressed as percentages for sensitivity, specificity, PPV, NPV, and accuracy. PI ≥ 2.530 demonstrated 100% sensitivity, 98.6% specificity, 95.2% PPV, 100% NPV, and 98.9% overall accuracy for predicting post-spinal hypotension. PPV, positive predictive value; NPV, negative predictive value; PI, perfusion index

Cut-off	Sensitivity	Specificity	PPV	NPV	Accuracy
≥2.530	100.0	98.6	95.2	100.0	98.9

## Discussion

The present study showed that approximately one-quarter of adult patients undergoing elective below-umbilical surgeries under spinal anesthesia developed hypotension. Baseline PI was significantly higher in patients who developed hypotension than in those who did not, whereas other baseline variables, including age, gender, BMI, ASA status, HR, and MAP, were comparable between the groups. Baseline PI also demonstrated excellent discriminatory ability, with an AUC of 0.999 and an optimal cut-off of 2.53, providing very high sensitivity and specificity. These findings suggest that PI may be a useful, non-invasive predictor of hypotension following spinal anesthesia.

Patodi et al. first demonstrated the usefulness of PI for predicting hypotension following spinal anesthesia in patients undergoing lower abdominal surgery, with ROC analysis showing significant predictive ability [1]. In a prospective observational study involving patients undergoing lower-segment cesarean section (LSCS), Inamanamelluri et al. similarly demonstrated a significant relationship between PI and intraoperative hypotension [2]. Duggappa et al. subsequently reported that baseline PI could predict hypotension following spinal anesthesia in parturients undergoing LSCS, further supporting its potential role as a preoperative hemodynamic predictor [3]. Lal et al. also demonstrated that pre-spinal PI was significantly different between patients who developed hypotension and those who remained hemodynamically stable following spinal anesthesia for cesarean delivery [4].

Further evidence has been provided by Pradhan et al., who demonstrated that PI could be used to predict hypotension following spinal anesthesia in LSCS, although the diagnostic threshold differed from that observed in the present study [5]. Jabarulla et al. similarly reported a significant association between baseline PI and the occurrence of hypotension following spinal anesthesia in parturients [6]. More recently, Bhat and Soori demonstrated the predictive usefulness of PI in patients receiving spinal anesthesia, further supporting its potential application beyond a single surgical population [7]. Harde et al. also found that higher baseline PI was associated with post-spinal hypotension in patients undergoing LSCS, reinforcing the relationship between peripheral perfusion and hemodynamic response to sympathetic blockade [8].

Raghavendran et al. reported that PI was a reliable tool for predicting hypotension during subarachnoid block for cesarean section [9]. Similarly, Singh et al. demonstrated that PI could be used as a predictor of hypotension during spinal anesthesia for LSCS [10]. Grewal et al. also reported significant predictive utility of PI for intraoperative hypotension following subarachnoid block in cesarean patients [11]. Mohan et al., in a prospective randomized study, further supported the role of pulse oximeter-derived PI as a predictor of hypotension following spinal anesthesia in LSCS [12]. Jana et al. demonstrated a significant relationship between PI and intraoperative hypotension during LSCS under spinal anesthesia, providing additional evidence for the association between peripheral perfusion and post-spinal hemodynamic changes [13]. Kesavankutty et al. evaluated the diagnostic accuracy of preoperative PI for predicting post-spinal hypotension and similarly found that baseline PI could provide useful discriminatory performance in parturients undergoing cesarean delivery [14]. Finally, Swarnakumar et al. demonstrated the predictive value of baseline PI in elderly patients undergoing lower limb orthopedic surgery, providing evidence that the usefulness of PI may extend beyond obstetric populations [15].

Taken together, these studies provide consistent evidence that a higher baseline PI is associated with an increased likelihood of hypotension following spinal anesthesia [15]. However, the reported cut-off values, sensitivity, specificity, and AUCs have varied considerably between studies. Such differences may reflect variations in patient characteristics, surgical populations, definitions of hypotension, spinal anesthetic dose, monitoring techniques, and hemodynamic management protocols [1-15]. The present study extends the existing evidence by demonstrating a strong predictive association between baseline PI and post-spinal hypotension in a mixed adult population undergoing elective below-umbilical surgeries rather than a predominantly obstetric population [1-15].

Physiologically, PI represents the ratio of pulsatile to non-pulsatile blood flow at the monitoring site and is influenced by peripheral vascular tone and microcirculatory perfusion. Peripheral vascular tone is substantially regulated by sympathetic activity. A higher baseline PI may therefore reflect relatively greater peripheral vasodilation and lower vasomotor tone. Following spinal anesthesia, sympathetic blockade produces vasodilation and a reduction in systemic vascular resistance. Patients with a higher baseline PI may consequently have less vascular reserve and may experience a greater reduction in blood pressure following sympathetic blockade [1-15]. The significant negative correlations observed between PI and MAP at several time points after spinal anesthesia in the present study support this proposed mechanism, with higher PI values being associated with lower MAP during the period when sympathetic blockade was evolving.

The findings are also consistent with previous studies demonstrating that patients with higher pre-spinal PI experience a greater fall in blood pressure and are more likely to require vasopressor intervention following subarachnoid block [1-15]. Thus, baseline PI may act as a simple surrogate marker of peripheral vascular tone and may help anesthesiologists anticipate an individual's hemodynamic response to spinal anesthesia.

From a clinical perspective, the very high AUC of 0.999 and the excellent sensitivity and specificity observed at the identified cut-off of 2.53 suggest that baseline PI could potentially be incorporated into routine pre-spinal assessment for risk stratification. Identification of patients at increased risk of hypotension could allow clinicians to institute appropriate preventive measures, including optimization of fluid therapy, early preparation or prophylactic use of vasopressors, closer hemodynamic monitoring, and, where appropriate, consideration of modifications to the anesthetic technique. This approach is particularly attractive because PI is obtained from a conventional pulse oximeter and therefore does not require additional invasive monitoring. It is continuous, non-invasive, and readily available, and requires minimal additional cost or effort [1-15].

Nevertheless, the variability in reported PI thresholds across the available literature should be considered. Different studies have identified different optimal cut-off values and diagnostic performances, even within similar obstetric populations [2-14]. This variability may result from differences in baseline patient characteristics, peripheral temperature, sympathetic tone, spinal anesthetic dose, definition of hypotension, timing of PI measurement, surgical stimulus, and hemodynamic treatment protocols. Therefore, although PI appears to be a promising predictor of spinal anesthesia-induced hypotension, a single universal cut-off should not be applied without appropriate external validation. The cut-off of 2.53 identified in the present study should consequently be interpreted in the context of the study population and anesthetic protocol.

The present study has several strengths, including its prospective design, standardized anesthetic technique, protocolized hemodynamic management, and serial measurement of PI and blood pressure during the first 30 minutes following spinal anesthesia. The inclusion of patients undergoing different types of elective below-umbilical surgery also provides evidence regarding the potential applicability of PI beyond obstetric populations, which have constituted a substantial proportion of previous studies [1-15].

However, several limitations should be acknowledged. The study was conducted at a single center with a modest sample size and purposive sampling, which may limit generalizability to other institutions and higher-risk populations. Only ASA I-II patients were included, while parturients, patients with major systemic disease, and patients with extreme PI values (<0.2 or >10) were excluded. Therefore, the predictive performance of PI in these populations remains uncertain. In addition, potential confounding factors such as ambient temperature, anxiety, peripheral vascular tone, and vasoactive medications, all of which can influence PI, were not formally quantified or controlled. Similar limitations have been recognized in previous observational studies evaluating PI as a predictor of post-spinal hypotension [1-15].

Future research should include larger, multicenter studies involving more diverse patient populations and should compare PI with other non-invasive predictors of spinal anesthesia-induced hypotension. Further investigation should also evaluate whether PI-guided prophylactic strategies, such as targeted fluid therapy or vasopressor administration, can reduce the incidence and severity of hypotension and improve clinical outcomes. Such studies would help determine whether the strong diagnostic performance of baseline PI observed in the present study translates into meaningful improvements in clinical practice [15].

## Conclusions

Baseline PI appears to be a useful, non-invasive predictor of hypotension following spinal anesthesia in patients undergoing elective below-umbilical surgery. The findings suggest that assessment of baseline PI before spinal anesthesia may help identify patients who are more likely to develop hemodynamic instability and may allow anesthesiologists to anticipate and prepare for hypotension through closer monitoring and timely hemodynamic management.

However, the findings should be interpreted within the context of the study population and setting. Further validation in larger, multicenter studies involving broader patient populations is warranted before the identified PI threshold can be routinely applied across different surgical and anesthetic settings. Future studies should also evaluate whether incorporating PI-based risk stratification into clinical practice and preventive hemodynamic strategies can reduce the incidence and clinical consequences of post-spinal hypotension.
